# Angina Bullosa Hemorrhagica: A Rare Presentation of Oral Bleeding

**DOI:** 10.7759/cureus.56797

**Published:** 2024-03-23

**Authors:** Jayant Singh, Anil Wanjari

**Affiliations:** 1 Department of Medicine, Jawaharlal Nehru Medical College, Datta Meghe Institute of Medical Science (Deemed to be University), Wardha, IND

**Keywords:** chronic steroid use, spontaneous resolution, oral mucosa, trauma, blood-filled blister

## Abstract

Angina bullosa hemorrhagica (ABH) is a rare condition seen in the oral cavity which is characterized by the presence of single or multiple blood-filled cavities which are generally not associated with any other systemic illness or condition. These lesions tend to rupture spontaneously and lead to epithelial erosions, which heal over the course of a few days without any signs of scarring. The condition is mostly attributed to trauma in the oral cavity, which occurs as a result of sharp metallic crowns or other such metal implants on the teeth or due to chewing hard and crispy food. This report presents a case of a 50-year-old female with no comorbidities who presented with bleeding from the mouth after eating cashew nuts. The case was diagnosed clinically and conservatively managed. The report aims to increase awareness regarding the causes and management of the condition.

## Introduction

Angina bullosa hemorrhagica (ABH) is a rare benign condition that is characterized by the presence of blood-filled lesions in the oral cavity, which, while primarily painless, may be painful in some cases and resolve spontaneously by bursting in a short time and subsequently heal without scarring [1].

The prevalence of ABH is reported to be around 0.05% in oral pathology centers and departments of oral medicine, generally seen in patients over the age of 30, with a peak of incidence in patients in the fifth decade of life with no apparent gender preference [2]. A Brazilian study reported a prevalence of 0.18% in 12,727 patients presenting with oral and maxillofacial lesions over 14 years from 2006 to 2020 [3]. Though there is no gender predilection, as mentioned above, a study by da Rosa et al. proclaimed a slightly greater female predilection, which was shown to be 55.3% [4].

The etiopathogenesis of this condition has been linked to trauma to the oral mucosa by hard or crispy food, most commonly in the soft palate. However, other sites such as the anterior pillar of the fauces, hard palate, arytenoids, epiglottis, esophagus, and pharyngeal wall may also be involved [5]. This condition is also associated with chronic use of steroid inhalers and systemic conditions such as diabetes mellitus and hypertension [4,6]. These lesions have been shown to resolve spontaneously by removing the stimulus causing the lesions [5]. This condition is not a very common occurrence in day-to-day practice; thus, documentation and reporting must be done to promote awareness and prevent unnecessary investigations.

## Case presentation

**Figure 1:**
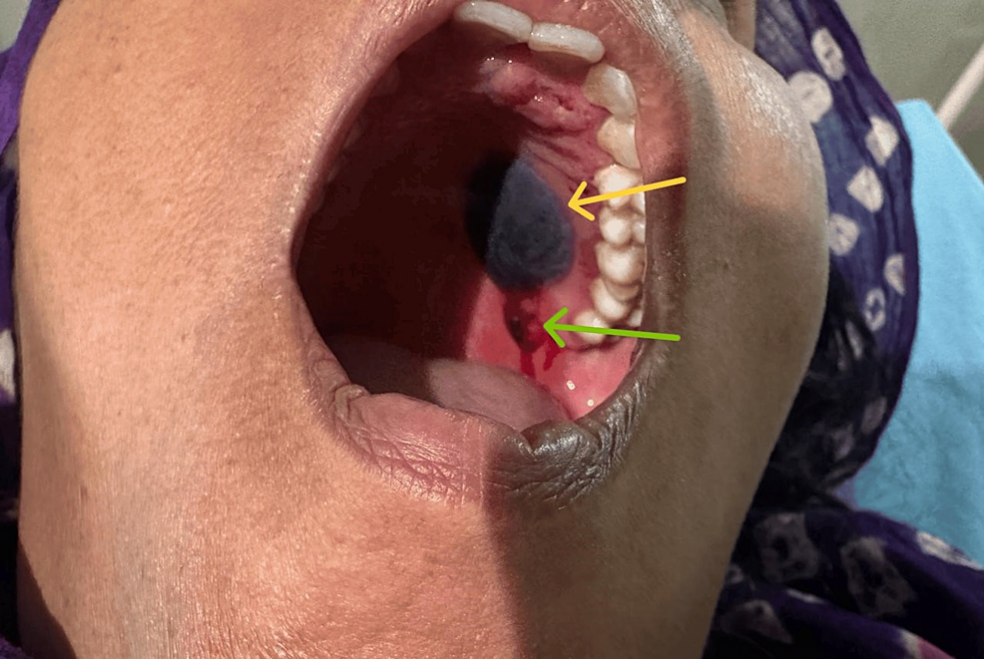
A single blood-filled lesion (yellow arrow) on the left side of the hard palate with blood oozing (green arrow)

A 50-year-old female presented to the medicine outpatient department with bleeding in the oral cavity. Following the onset of bleeding, she became aware of a large blister on the hard palate, which prompted her to seek medical attention. The patient gave a history of eating cashew nuts a few hours before the episode of bleeding started. On further inquiry, she reported that the lesion was painless, and she had no complaints of any choking sensation in the throat or difficulty in respiration. She also denied any history of diabetes mellitus, hypertension, or any other bleeding disorders running in the family, which might have led to a similar presentation. The patient also denied any history of spontaneous bleeding from the mouth in the past. On examination, a single, blood-filled blister was noticed on the hard palate on the left side. The blister was approximately 3 cm x 2 cm in size, smooth, and non-tender on palpation. There was also evidence of fresh, bright red color blood oozing from the inferior aspect of the lesion, as shown in Figure 1.

The patient was hemodynamically stable with a pulse of 88 beats per minute, blood pressure of 136/84 mm/Hg, temperature of 98^o^ Fahrenheit, saturation of peripheral oxygen (SpO2) 96%, and respiratory rate of 17 cycles/minute. The hematology report had no significant findings, with bleeding time being two minutes 45 seconds and clotting time being four minutes 30 seconds, both within normal limits. The platelet count was 209 x 10^9^/L, and the liver function test (LFT) was normal with an international normalized ratio (INR) of 1.3. The random blood glucose picture revealed a value of 135 mg/dl, which is normal.

The patient was admitted for observation and was provided conservative management with antibiotics, betadine mouth rinse, and multivitamins. The patient's condition improved following this line of treatment, and the lesion started to heal. There was no episode of fresh bleeding from the mouth in the following days. The patient was discharged on the fourth day and advised follow-up.

## Discussion

ABH, named such by Badham in 1967, is a misnomer as it is not actually associated with angina; instead, it is so called as these blisters, upon enlarging, can cause a choking sensation in the patient [7]. Another more appropriate term to describe this condition is traumatic oral hemophlyctenosis, which represents the same lesion with an underlying traumatic origin [8].

Though the exact mechanism of the formation of these blisters remains unclear, factors like oropharyngeal trauma secondary to eating hard and crispy food, hypertension, diabetes mellitus, and a history of chronic inhaled steroid usage are frequently associated with this disorder. In a study by Grinspan et al., it was reported that out of 54 patients, 24 patients (44.4%) had a history of diabetes mellitus or a family history of hyperglycemia [9]. It is proposed that vascular fragility may be a cause of bleeding in the case of diabetes mellitus [10]. Chronic inhaled steroid use has been associated with causing ABH, owing to the atrophy of the epithelium and changes in the elastic fibers of lamina propria, which predispose to the formation of hemorrhagic bullae following trauma [11]. In the case of hypertension, though, the exact mechanism remains unclear.

In the current patient, the only risk factors were the age of the patient and a recent history of oropharyngeal trauma secondary to eating cashew nuts, with blood pressure and random blood sugar being normal and no history of chronic inhaled steroid use for any relevant condition. The criteria used to diagnose ABH were described by Ordioni et al., which are as follows: (i) A clinically evident hemorrhagic blister or erosion in the oral mucosa with a history of bleeding, (ii) localization is exclusively oral or oropharyngeal, (iii) palatal localization, (iv) triggering event such as intake of food, (v) recurrent lesions, (vi) healing within a few days without scarring, (vii) lesion is painless, (viii) platelet count and coagulation parameters are normal, (ix) direct immunofluorescence is negative. Out of these, if six or more than six are met, the patient can be diagnosed as a case of ABH [12]. In this patient, seven out of the given nine criteria were met, therefore making the diagnosis of ABH highly likely.

The diagnosis of ABH is primarily a diagnosis of exclusion, which is arrived at after eliminating the possibilities of other disorders that might present with similar complaints, such as thrombocytopenia, Behcet’s disease, and von Willebrand disease [13-15]. Therefore, it is crucial to order blood investigations to check for any abnormalities in the blood picture in terms of platelet count, bleeding time, clotting time, and other such hematological parameters, which in this patient turned out to be normal.

In the current case, the management, as mentioned earlier, was done using antibiotics, betadine gargles, and multivitamins. The main aim of this line of treatment was to prevent any secondary infections in the patient. Since oropharyngeal trauma is the suspected cause of bleeding in such patients, there are chances of bacterial infections of the oral cavity due to the disruption of the mucosal lining by organisms such as *Streptococcus* and *Neisseria* [16]. Also, many deoxyribonucleic acid (DNA) and ribonucleic acid (RNA) viruses also have the potential to infect the oral mucosa in conditions with compromised immunity [17]. Multivitamins on the other hand act as immunity boosters, thus also playing a role in enhancing the immune functions [18]. A similar management strategy has been elicited in other cases of ABH by other practitioners [19,20].

An interesting point to be noted in the treatment of ABH is the role of steroids. Though steroids were not used in the management of this patient, a study published by Okobi et al. demonstrates the effectiveness of using methylprednisolone IV stat in the treatment of an acute case of ABH with an expanding blood-filled blister that could potentially cause respiratory tract obstruction in the patient [5]. The patient was reported to have marked improvement in his condition following the administration of methylprednisolone, which was attributed to the anti-inflammatory properties of steroids. This, however, as aptly put forward by Okobi et al. in their report, seemed to point toward an inflammatory pathology as being the root cause for the formation and progression of the condition and not just simple oropharyngeal trauma [5]. In either case, however, steroids have shown contrasting action acting as both the cause and the cure for this condition, which warrants further research into the efficacy and potential deleterious effects of using steroids in this condition. 

## Conclusions

ABH is a rare condition seen in middle-aged and elderly patients and is a diagnosis of exclusion. Though not a fatal condition, it is important to rule out any other bleeding pathologies or autoimmune involvement by thorough hematological examination, and a history of hypertension, diabetes mellitus, and long-term steroid inhaler use is to be ruled out to prevent any instances of unnecessary investigations.

As for the management, the patient can be treated conservatively with antibiotics and multivitamins to prevent any secondary infections. Though steroid use can also help to resolve the lesions, it should be used with caution due to the lack of definite research greenlighting its use.
